# Crystal structure of *Saccharomyces cerevisiae* mitochondrial GatFAB reveals a novel subunit assembly in tRNA-dependent amidotransferases

**DOI:** 10.1093/nar/gku234

**Published:** 2014-04-01

**Authors:** Yuhei Araiso, Jonathan L. Huot, Takuya Sekiguchi, Mathieu Frechin, Frédéric Fischer, Ludovic Enkler, Bruno Senger, Ryuichiro Ishitani, Hubert D. Becker, Osamu Nureki

**Affiliations:** 1 Unité Mixte de Recherche 7156 Génétique Moléculaire Génomique Microbiologie, Centre National de la Recherche Scientifique, Université de Strasbourg, F-67084 Strasbourg, France; 2 Department of Biophysics and Biochemistry, Graduate School of Science, The University of Tokyo, Bunkyo-ku, 113-0033 Tokyo, Japan; 3 Institute of Molecular Life Sciences, University of Zurich, CH-8057 Zurich, Switzerland; 4 Unité Propre de Recherche Architecture et Réactivité de l’ARN, Centre National de la Recherche Scientifique, Institut de Biologie Moléculaire et Cellulaire, Université de Strasbourg, F-67084 Strasbourg, France

## Abstract

Yeast mitochondrial Gln-_m_tRNA^Gln^ is synthesized by the transamidation of mischarged Glu-_m_tRNA^Gln^ by a non-canonical heterotrimeric tRNA-dependent amidotransferase (AdT). The GatA and GatB subunits of the yeast AdT (GatFAB) are well conserved among bacteria and eukaryota, but the GatF subunit is a fungi-specific ortholog of the GatC subunit found in all other known heterotrimeric AdTs (GatCAB). Here we report the crystal structure of yeast mitochondrial GatFAB at 2.0 Å resolution. The C-terminal region of GatF encircles the GatA–GatB interface in the same manner as GatC, but the N-terminal extension domain (NTD) of GatF forms several additional hydrophobic and hydrophilic interactions with GatA. NTD-deletion mutants displayed growth defects, but retained the ability to respire. Truncation of the NTD in purified mutants reduced glutaminase and transamidase activities when glutamine was used as the ammonia donor, but increased transamidase activity relative to the full-length enzyme when the donor was ammonium chloride. Our structure-based functional analyses suggest the NTD is a trans-acting scaffolding peptide for the GatA glutaminase active site. The positive surface charge and novel fold of the GatF–GatA interface, shown in this first crystal structure of an organellar AdT, stand in contrast with the more conventional, negatively charged bacterial AdTs described previously.

## INTRODUCTION

The existing paradigm for mitochondrial aa-tRNA synthesis in eukaryotes cannot explain the translation of glutamine codons (1). All eukaryotic genomes analyzed so far only encode a single cytosolic glutaminyl-tRNA synthetase (_c_GlnRS). The gene encoding an organellar GlnRS is always missing (2), but its absence is compensated by the so-called transamidation pathway, which also generates mitochondrial glutaminyl-tRNA^Gln^ (Gln-_m_tRNA^Gln^). As most bacteria and all known archaea use this pathway to generate Gln-tRNA^Gln^, its presence in mitochondria probably reflects the endosymbiotic origin of these organelles (3–5). In this transamidation pathway, the mischarged glutamyl-tRNA^Gln^ (Glu-tRNA^Gln^) is formed by a non-discriminating glutamyl-tRNA synthetase (ND-GluRS), and then is converted into Gln-tRNA^Gln^ by a tRNA-dependent amidotransferase (AdT).

The formation of Gln-_m_tRNA^Gln^ has only been examined in a few species: some may import _c_GlnRS (6,7), while others may use GatCAB and the transamidation pathway (8,9). Our recent study revealed that yeast Gln-_m_tRNA^Gln^ is synthesized by a transamidation pathway involving a novel heterotrimeric AdT named GatFAB (10). It transamidates Glu-_m_tRNA^Gln^ generated by an imported pool of cytoplasmic GluRS (_c_GluRS), which acts as a ND-GluRS in the mitochondrion (10).

The GatA and GatB subunits of GatFAB are homologous to those of bacterial and eukaryotic GatCABs. GatA hydrolyzes Gln into Glu and an ammonia molecule, which travels to GatB through an intramolecular ammonia tunnel (11,12). Upon reaching the GatB active site, it reacts with γ-phosphoryl-glutamyl-_m_tRNA^Gln^ formed by the ATP-dependent activation of the Glu side-chain carboxyl group. These reactions are coupled, as shown by activation of the GatA glutaminase activity upon binding of Glu-tRNA^Gln^ and ATP by GatB (13).

The third subunit, GatF, is a new type of AdT subunit found only in fungal genomes. It is present in the place of the GatC found in bacterial, plant and mammalian AdTs, where it reinforces the interaction between the GatA and GatB subunits by encircling their interface (14–18). Given this pivotal role, it is surprising that GatC and GatF share poor sequence homology and that GatF is approximately twice the size of any GatC, although both subunits are comparatively smaller than the other catalytic subunits.

Our crystal structure of GatFAB from *Saccharomyces cerevisiae*, at 2.0 Å resolution, is the first AdT structure from a eukaryotic organelle. A structural comparison between GatF and GatC revealed that it has an N-terminal extension domain (NTD), which forms an extended interface with GatA. Our structure shows that GatF is a functional homolog of GatC based on its role in maintaining the GatFAB trimer assembly. However, this role is achieved using a different architecture from that of GatC, since the NTD of GatF extends to the glutaminase site of GatA. To understand the functional properties of the NTD of GatF, we generated structure-based NTD deletion mutants and analyzed their enzymatic activities and ability to complement a yeast Δ*gatF* strain. Our results show that deletions of the NTD impacted cell growth, especially in respiration, but were not lethal. The ability to hydrolyze glutamine and to use the resulting ammonia for Gln-_m_tRNA^Gln^ synthesis was reduced in GatFABs assembled with these GatF mutants. However, for one such mutant, transamidation with NH_4_Cl as the ammonia donor was enhanced relative to the full-length enzyme. These results suggest that truncation of the NTD alters or destabilizes the GatA active site. Thus, we propose that the GatF NTD is present to modulate folding and stability of the GatA core, while the GatC-like portion of GatF serves to maintain the GatA–GatB assembly.

## MATERIALS AND METHODS

### Protein purification

The yeast *gatFAB* operon was designed as described previously (10). The putative mitochondrial target sequences in the N-termini of GatB (residues 1–15) and GatF (residues 1–23) were removed. The *gatFAB* operon was subcloned into a modified pCGFP-BC vector containing a C-terminal His-tag and a Tobacco Etch Virus (TEV) protease cleavage site for the tag removal (19). To facilitate the structural study, we truncated the C-terminal helical (residues 330–474) and YqeY (residues 475–541) domains (GatFABΔHY), which showed high flexibilities in the reported bacterial GatCAB structures (17,18). To overproduce GatFABΔHY, the recombinant plasmid was transformed into *Escherichia coli* BL21 (DE3) Star2 CodonPlus (Stratagene). The cells were grown to an absorbance at 600 nm of 0.5 and gene expression was induced with 0.1 mM isopropyl-β-D-thiogalactopyranoside (IPTG), followed by incubation at 18°C for 18 h. After centrifugation at 5000 ×g for 5 min, the harvested cells were suspended in 50 mM Tris-HCl buffer (pH 7.0) containing 250 mM NaCl, 5 mM β-mercaptoethanol, 5% glycerol and 0.1 mM phenylmethylsulfonyl fluoride, and then were disrupted by sonication. The supernatant after centrifugation was loaded on a Ni-nitrilotriacetic acid (NTA) agarose (QIAGEN) column and eluted with 500 mM imidazole. The eluted fraction was treated with TEV protease and reloaded onto the Ni-NTA column to remove the His-tag. The protein was loaded onto a HiTrap Heparin HP (GE healthcare) column and eluted with a linear gradient of 200–1000 mM NaCl. The recombinant GatFABΔHY was finally purified with a Hiload 16/600 Superdex200 gel filtration column (GE healthcare) with the buffer containing 20 mM Tris-HCl buffer (pH 8.0), 100 mM NaCl and 1 mM dithiothreitol. The fractions were concentrated to 10 mg/ml.

For the biochemical assays, GatFABΔY was overexpressed with the same procedure as GatFABΔHY. However, GatFAB_F24–183_ (pseudo-wild-type) and the series of GatF mutants (GatFAB_F37–183_, GatFAB_F47–183_, GatFAB_F58–183_ and GatFAB_F67–183_) were expressed by autoinduction in 200 ml ZYP-5052 medium (20) or addition of 10% (w/v) glucose together with IPTG in LB medium. GatFAB pseudo-wild-type, GatFABΔY and GatFABΔHY were purified with Ni-NTA agarose (QIAGEN) and Superdex200 10/300 column (GE Healthcare). The other GatF mutants were purified with MagneHis (Promega).

### Western blots quantification of expression levels of GatF mutants

The GatF mutants were overexpressed by autoinduction in 200 ml ZYP-5052 medium with strong agitation (20). The cells were grown to 0.1 O.D._600 nm_ and then incubated at 20°C for 32 h (total 36 h). Each cell pellet (approximately 5 g on average) was resuspended in 10 ml lysis buffer (50 mM Tris-HCl pH 7.8, 500 mM NaCl, Mini EDTA-free protease inhibitor cocktail, Roche) and disrupted by sonication. The supernatant after centrifugation was recovered and aliquoted, and the aliquots were flash frozen in liquid nitrogen and then kept at −80°C. The purified pseudo-wild-type GatFAB_F24–183_ protein (2.4 μg) and total protein (30 μg) from each extract were loaded on 15% SDS-PAGE gel. Electrophoresis was stopped when the migration front almost reached the bottom of the gel. The gel was transferred onto a Transblot Turbo Pack membrane (PVDF). Mouse anti-His-tag primary antibodies (1:1000) were used to quantify the amount of GatB in each extract. Detection was carried out using Horseradish peroxidase (HRP)-conjugated goat anti-mouse antibodies (1:2000).

### Crystallization and data collection

Crystallization was performed at 20°C by the sitting drop vapor diffusion method. The crystals of GatFABΔHY free form were grown within 2 days by mixing equal volume of the 10 mg/ml GatFABΔHY protein solution and the reservoir solution containing 50 mM sodium malonate, 200 mM Mg(NO_3_)_2_ and 10–13% PEG3350. The Gln-bound form of GatFABΔHY was prepared by mixing the protein solution with glutamine to a final concentration of 10 mM before crystallization. The SeMet-labeled protein was crystallized under the same conditions as those for the native protein. The crystals were cryoprotected in the reservoir solution supplemented with 20% (v/v) 2-methyl-2,4-pentanediol and flash-cooled in a nitrogen stream at 100 K. Diffraction data were collected at beamlines NW12A and NE3A at KEK PF-AR (Tsukuba, Japan) and BL41XU at SPring-8 (Harima, Japan). Diffraction data were processed using the program HKL2000 (21).

### Structure determination and refinement

Although *S. cerevisiae* GatA and GatB show high sequence similarity with their bacterial counterparts, our attempts to solve the structure by molecular replacement were unsuccessful. Therefore, the structure was determined by the multiwavelength anomalous dispersion method. The 10 selenium sites were initially identified by the program SHELEXC and SHELEXD with the dataset collected at the peak wavelength (22). Subsequent refinements of the heavy atom parameters and phase calculations were performed with the program SHARP (23), followed by density modification and automated model building with the program RESOLVE (24). The resulting initial model was manually improved to fit into the electron density map using the program COOT (25). Eventually, the atomic models of the apo and Gln-bound form of GatFABΔHY were refined against the native datasets up to 2.0 Å resolutions, respectively, using the program PHENIX (26). Molecular graphics were illustrated with CueMol2.0 (http://www.cuemol.org/).

### Construction of tRNA docking model

In order to build a model of the C-terminal domains of GatB, we superposed the GatB subunit of *Thermotoga maritima* GatCAB-tRNA^Gln^-GluRS complex structure and placed the YqeY and helical domains of *T. maritima* GatB in the C-terminus of *S. cerevisiae* GatB (18). Since bacterial tRNA^Asn^ was shown to be an efficient substrate for GatFAB (10), we then superposed the GatB subunit and tRNA^Asn^ of *Thermus thermophilus* GatCAB-tRNA^Asn^-AspRS complex structure and modeled the tRNA^Asn^ onto the *S. cerevisiae* GatFAB structure (17).

### Complementation of yeast Δ*gatB* and Δ*gatF* strains

The *gatB* and *gatF* genes (*PET112* and *GTF1*) were subcloned into pRS316 vector under the control of their endogenous promoters. Yeast Δ*gatB* and Δ*gatF* strains (Euroscarf) were transformed with these plasmids to obtain *gatB* and *gatF* shuffle strains in which a plasmid-borne wild*-*type gene complements the chromosomal deletion. The genes encoding *gatB* and *gatF* mutants were subcloned into p415MDH vector (10). The GatB and GatF mutants were overexpressed under the control of the mitochondrial malate dehydrogenase (MDH) promoter. All mutants were fused with the N-terminal canonical mitochondrial target sequence of MDH (10). The *gatB* and *gatF* shuffle strains were transformed with these plasmids, respectively. As a negative control, the strain was transformed with an empty vector. Then, these strains were plated on 5-Fluoroorotic acid plates to select for loss of the plasmid encoding wild-type genes.

### Transamidation assay

To characterize the transamidation and glutaminase activities of the GatF mutants, we used Glu-tRNA^Gln^ from unfractionated yeast tRNA enriched to 20% tRNA^Gln^ and yeast _c_GluRS. To measure the transamidation activity of the GatB mutants, *Helicobacter pylori* tRNA^Gln^ (over 70% purity) was used, this tRNA having the U1-A72 base pair and small D-loop identity determinants of trimeric AdTs found on yeast _m_tRNA^Gln^ (27).

*H. pylori* tRNA^Gln^, yeast unfractionated tRNA, *H. pylori* GluRS2 (ND-GluRS) and yeast _c_GluRS were overproduced and purified as described in previous studies (10,28). The aminoacylation mixture (100 μl) containing 100 mM Na-HEPES (pH 7.2), 30 mM KCl, 10 mM ATP, 12 mM MgCl_2_, 12 μM [^14^C]L-glutamate ([^14^C]Glu) (330 cpm/pmol, Amersham), 0.1 mg/ml BSA, 10 μM *H. pylori* tRNA^Gln^ or yeast unfractionated tRNA and 5 μM *H. pylori* GluRS2 or yeast _c_GluRS was incubated at 37°C for 30 min. The [^14^C]Glu-tRNA^Gln^ was extracted using acid-buffered phenol and chloroform and precipitated with ethanol (28). The standard transamidation mixture containing 100 mM Na-HEPES (pH 7.2), 30 mM KCl, 12 mM MgCl_2_, 10 mM ATP, 1 mM L-Gln as amide group donor, approximately 6 μM [^14^C]Glu-tRNA^Gln^ and 0.1–0.2 μM *S. cerevisiae* GatFAB variants was incubated for 10–20 min at 37°C. For the transamidation tests performed without Gln, 20 mM NH_4_Cl was used. Activities were then assayed as previously described (28).

### Glutaminase assay

The glutaminase mixture containing 100 mM Na-HEPES (pH 7.2), 30 mM KCl, 12 mM MgCl_2_, 10 nM ATP, 50 μM [^14^C]L-glutamine ([^14^C]Gln), 0.025 mg/ml BSA, approximately 6 μM Glu-tRNA^Gln^ and 0.1 μM *S. cerevisiae* GatFAB variants was incubated at 37°C for 10–20 min. The reaction was stopped and spotted on Thin-layer Chromatography (TLC) plates as above.

## RESULTS

### Structure determination

**Figure 1.**
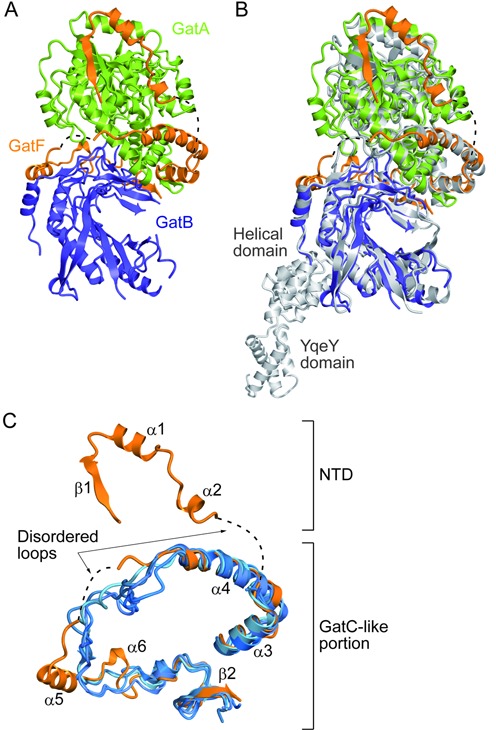
Structural comparison of GatFAB with GatCAB. (**A**) Ribbon representation of the crystal structure of *S. cerevisiae* GatFAB, consisting of the GatA (green), GatB (navy blue) and GatF (orange) subunits. Dashed lines indicate the disordered loops of GatF (residues 59–66 and 114–128). (**B**) The superposition of the crystal structure of *S. cerevisiae* GatFAB onto that of *S. aureus* GatCAB. GatFAB is shown in the same color code as in (A). *S. aureus* GatCAB is colored gray (PDB ID: 3IP4). (**C**) Structural comparison of *S. cerevisiae* GatF with the bacterial GatC. GatF is colored orange. The *T. thermophilus, T. maritima, S. aureus* and *A. aeolicus* GatCAB structures (PDB IDs: 3KFU, 3AL0, 3IP4 and 3H0R) are colored blue and superposed onto that of *S. cerevisiae* GatFAB.

To facilitate crystallization, the putative N-terminal mitochondrial targeting sequences of GatB (residues 1–15) and GatF (residues 1–23) were eliminated. Furthermore, we truncated the C-terminal helical and YqeY domains (residues 330–474 and 475–541, respectively), which were reported to be highly flexible (17,18). The crystal structure of the heterotrimeric GatFAB AdT from *S. cerevisiae* was determined by the multiple wavelength anomalous diffraction method. The final models of the free and glutamine-bound forms were refined at 2.0 Å resolutions to free *R*-factors of 22.2 and 21.9%, respectively (Figure 1A, Tables 1 and 2).

**Table 1:** Data collection and phasing statistics

	SeMet		Free form	Gln-bound form
Data collection				
X-ray source	SPring-8 BL41XU		PF-AR NE3A	PF-AR NW12
Wavelength (Å)	0.97914 (peak)	0.97937 (inflection)	1.0	1.0
Space group	*P*2_1_2_1_2_1_			
Cell dimensions				
*a*, *b*, *c* (Å)	61.78, 85.35, 189.58	61.80, 85.43, 189.57	61.95, 86.30, 191.23	62.44, 85.43, 194.78
α, β, γ (°)	90, 90, 90	90, 90, 90	90, 90, 90	90, 90, 90
Resolution (Å)	50.0–2.10	50.0–2.10	50.0–1.95	50.0–2.0
	(2.14–2.10)	(2.14–2.10)	(1.98–1.95)	(2.03–2.0)
*R*_merge_ (%)	10.1 (35.2)	9.6 (37.9)	11.8 (38.9)	7.1 (33.2)
*I*/σ(*I*)	82.19 (9.29)	54.34 (4.44)	21.38 (2.59)	36.15 (4.62)
Completeness (%)	99.9 (99.8)	99.6 (95.9)	98.7 (98.8)	96.9 (97.5)
Redundancy	23.4 (16.2)	11.2 (5.7)	5.3 (4.1)	5.3 (4.2)

*Highest resolution shell is shown in parentheses.

**Table 2:** Structure refinement statistics

	Free form	Gln-bound form
Refinement		
Resolution (Å)	50–1.95 (1.98–1.95)	50–2.0 (2.02–2.0)
No. of reflections	74 447	68 955
*R*_work_/*R*_free_	0.1881/0.2218	0.1890/0.2192
	(0.2715/0.2860)	(0.2735/0.3030)
No. of atoms		
Protein	6597	6596
Ions/Ligand	4	13
Water	274	327
Average *B*-factors (Å^2^)		
Protein	40.1	51.4
Ions/Ligand	45.5	41.1
Water	36.9	41.8
Coordinates error (Å)	0.52	0.50
r.m.s. deviations		
Bond lengths (Å)	0.004	0.006
Bond angles (º)	0.892	1.060

*Highest resolution shell is shown in parentheses.

### Overall structure

The present structure revealed that the organization of the yeast GatA and GatB subunits is quite similar to those of their bacterial homologs from *T. thermophilus, T. maritima, Staphylococcus aureus* and *Aquifex aeolicus* (14–18) (Figure 1B). The root mean square deviations between the GatA and GatB subunits of *S. cerevisiae* and those of *A. aeolicus* are 1.6 and 1.1 Å, respectively.

Like the bacterial GatCs, the GatF subunit is an unstructured protein (Figure 1C). Based on the superposition of our structure with that of the bacterial GatCAB, the yeast GatF can be divided into two halves, the C-terminal GatC-like portion and the N-terminal appended domain (NTD). Despite the low sequence similarity between GatF and GatC, the C-terminal part of GatF adopts a structure similar to that of the bacterial GatC, and surrounds the contact area between GatA and GatB (14). The NTD extends the interaction with GatA and encircles the amidase site. This structural property was not observed in any bacterial GatCAB structures (Figure 1B and C), and was not predicted in a recently proposed homology model for GatFAB (29).

### Two catalytic sites connected by an ammonia tunnel

**Figure 2.**
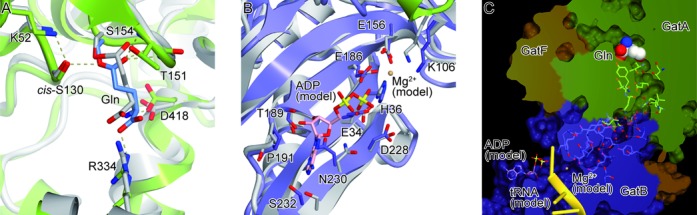
Active sites of GatFAB. GatFAB is colored as in Figure 1. (**A**) The glutaminase site of GatA in the Gln-bound form. The bound Gln molecule is shown as blue sticks. The crystal structure of *A. aeolius* GatA is colored gray and superposed onto that of *S. cerevisiae* GatA. (**B**) The amidotransferase site of GatB in the apo form. The crystal structure of *S. aureus* GatB is colored gray and superposed onto that of *S. cerevisiae* GatB. The modeled ADP molecule and Mg^2+^ ion are shown as pink sticks and as a sphere, respectively. (**C**) Surface model representation of a hydrophilic NH_3_ tunnel. The tunnel is filled with water molecules (red spheres), which interact with conserved residues of GatA and GatB. The modeled ADP and Mg^2+^ ion are shown as in (B). The modeled *T. thermophilus* tRNA^Asn^ is colored yellow.

The GatFAB AdT has two distinct and spatially separated catalytic sites for the glutaminase and transamidase reactions (Figure 2A and B). The present Gln-bound form structure reveals that the glutamine molecule is bound to the glutaminase site of GatA in a manner conserved among the bacterial homologs (14,15) (Figure 2A). The amide group of Gln is recognized by D418 and the carboxyl group is stabilized by R334. The Gln side chain is in the vicinity of the conserved S154-*cis*-S130-K52 catalytic scissors, which are thought to form the acyl intermediate at nucleophilic S154 like in the bacterial GatA structures (14,15).

In contrast, we were not able to identify any substrate in the AdT site of GatB, whereas *S. aureus* and *A. aeolius* GatB structures co-crystallized with ATP or its analogues (14,15) (Figure 2B). In order to analyze the transamidation mechanism, a structural comparison with *S. aureus* GatCAB allowed us to construct a model in which ADP and the catalytically essential Mg^2+^ ion are docked into the yeast GatB active site (Figure 2B). The Mg^2+^ ion plays a catalytic role in the nucleophilic reaction to form γ-phosphoryl-glutamyl-tRNA^Gln^ (14,15). Our model suggested that the ATP molecule can be bound through electrostatic interactions by the conserved E34, T189, N230 and S232 on the β1 and β11 strands in the bottom of the cradle domain, as in bacterial GatB. The model also indicated that the conserved H36, E156 and E186 recognize the Mg^2+^ ion.

Adjacent to the modeled Mg^2+^ ion is a molecular tunnel that penetrates the GatA and GatB subunits and connects the GatA glutaminase and GatB transamidase sites like in bacterial GatCABs (14) (Figure 2C). The tunnel is composed of hydrophilic residues that are highly conserved from bacteria to eukaryotes. Inside this tunnel are several electron densities corresponding to water molecules, or possibly ammonia. These structural features indicate that the GatFAB AdT harbors the same ammonia channeling system as bacterial AdT.

### The C-terminal domains of GatB play a key role in tRNA anchoring

**Figure 3.**
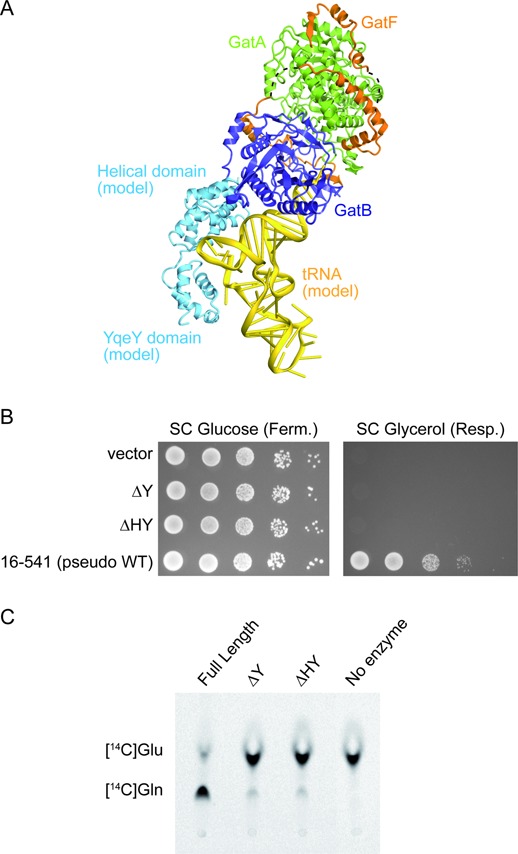
Functional analyses of YqeY and the helical domain of GatB. (**A**) Model of the GatFAB-tRNA complex. tRNA^Asn^ from *T. thermophilus* (PDB ID: 3KFU) and the helical and YqeY domains of GatB from *T. maritima* (PDB ID: 3AL0) are docked onto the GatFABΔHY structure. The modeled tRNA^Asn^ and the C-terminal domains of GatB are colored yellow and cyan, respectively. GatFAB is colored as in Figure 1. (**B**) Growth phenotypes of GatB mutants on fermentative (SC Glucose) or respiratory (SC Glycerol) media. (**C**) Bioimager scan of the TLC plate for a transamidase assay, which shows the absence of conversion of *H. pylori* tRNA^Gln^-bound [^14^C]Glu into [^14^C]Gln, catalyzed by the GatFAB mutants with the C-terminal deletions of GatB (ΔY and ΔHY).

To elucidate the tRNA binding mode of GatFAB, we performed a molecular superposition using the tRNA-bound structures of the bacterial GatCABs to dock the tRNA^Asn^ and the missing C-terminal helical and YqeY domains onto the present GatFAB structure (17,18). It has been shown that GatFAB efficiently transamidates a heterologous bacterial aspartyl-tRNA^Asn^ (Asp-tRNA^Asn^), and that tRNA^Asn^ and tRNA^Gln^ share the identity elements (10,27). The good fit between the modeled tRNA^Asn^ and GatB allowed the CCA terminus to contact the transamidation site (Figure 3A). Our docking model suggested that the cradle domain can recognize the U1-A72 base pair in the acceptor stem, which is the major determinant for transamidation by AdTs (17,18,27), and that the helical and YqeY domains bind the D-loop of tRNA (Figure 3A). To ascertain the importance of the interaction between the C-terminal domains of GatB and _m_tRNA^Gln^, we constructed C-terminal deletion mutants of GatB lacking the YqeY domain (GatBΔY, residues 16–474) and both the helical and YqeY domains (GatBΔHY, residues 16–329). As a positive control, we also constructed the GatB variant containing both C-terminal domains (GatB_16–541_, residues 16–541). Since all of the GatB mutants lack the endogenous N-terminal putative Mitochondrial Targeting Signal (MTS, we fused the MTS of the yeast MDH to the N-terminus of each GatB mutant to allow the *in vivo* mitochondrial import of these GatB variants. Indeed, GatB_16–541_ did not exhibit any growth defects in either fermentation or respiration, showing that the MTS of MDH suffices for import of GatB (Supplementary Figure S1A). *In vivo* complementation tests revealed that the strains expressing either GatBΔY or GatBΔHY displayed severe growth defects under respiratory conditions as compared to pseudo-wild-type GatB_16–541_, but had almost no impact on growth in fermentable medium (Figure 3B).

To characterize the enzymatic activities of these GatB mutants, we subsequently performed transamidation tests with the purified full-length and C-terminal deletion mutants of GatFAB (GatFABΔY and GatFABΔHY) using a heterologous bacterial Glu-tRNA^Gln^ possessing all the necessary identity determinants to be fully competent for transamidation assays (18). In the activity tests, the full-length GatFAB protein efficiently transamidated the bacterial Glu-tRNA^Gln^ into Gln-tRNA^Gln^, while the GatFABΔY and GatFABΔHY mutants displayed no measurable activities (Figure 3C).

These results demonstrate that GatB is mandatory for mitochondrial activity and that removal of the small YqeY domain consisting of only four helices inactivates GatB. Therefore, we propose that, like in the bacterial GatCAB (14,16), the helical and YqeY domains are critical for adequate anchoring of the Glu-_m_tRNA^Gln^ core and synthesis of Gln-_m_tRNA^Gln^.

### The GatC-like portion encircles the GatA–GatB interface

In the GatC-like portion of GatF, the α3 and α4 helices bind to GatB and GatA through hydrophobic and electrostatic interactions. These helices are also observed in the bacterial GatC structures (Figure 1B and C), α3 and α4 having ∼40% sequence similarity and ∼20% sequence identity with the corresponding *S. aureus* helices (10,30). The contact region of GatF is mainly composed of residues that are highly conserved in the fungal GatF proteins. Among these conserved residues, L77, L80 and L83 of helix α3, and L97, L101 and F103 of helix α4 are also conserved in the bacterial and mammalian GatC proteins. These residues mediate the hydrophobic interactions with GatA to form a helical bundle that stabilizes the heterotrimer (Supplementary Figure S2A). This domain is extended by an unstructured internal loop that crosses over the GatA–GatB contact area. The C-terminus of the β2 strand forms an antiparallel β-sheet with a β hairpin of GatB (Supplementary Figure S2D). These binding modes resemble those of the corresponding regions of the bacterial GatC. However, the internal loop of GatF structurally differs from that of GatC in several ways (Figure 1C, Supplementary Figure S2B and C). After the helical bundle, GatF possesses a flexible disordered loop (114–128), while GatC tightly contacts GatA (Supplementary Figure S2B). Moreover, GatF has two insertion helices, α5 and α6. The first insertion helix, α5, is anchored to GatB through hydrophobic interactions (Supplementary Figure S2C). Meanwhile, the bacterial GatC harbors the invariant Arg and Asp residues (R64 and D66 in *S. aureus* GatCAB) that interact with the conserved Ser and Asn residues of GatB (S19 and N52 in *S. aureus* GatCAB) (14), which are not conserved in yeast GatFAB (Supplementary Figure S2C).

### The NTD of GatF is bound to the GatA core domain

**Figure 4.**
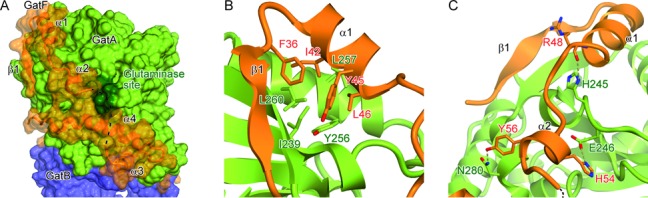
Interface between GatA and the NTD of GatF. GatFAB is colored as in Figure 1. (**A**) Surface representation of GatFAB. GatF is also shown in a ribbon representation. The glutaminase site is colored dark green. (**B** and **C**) Close-up views of the GatF–GatA interface.

The NTD is tightly bound to the GatA core domain and is composed of one β-strand, two α-helices and unstructured loop regions (Figures 1C and 4A). The linker loop connecting the NTD and the C-terminal GatC-like structure is completely disordered, reflecting the flexibility of the NTD (Figure 1A and C).

The first β strand of GatF folds into a twelve-stranded β-sheet central domain, together with eleven β-strands of GatA. In the β1 strand and α1 helix of GatF, F36, I42, Y45 and L46 form a hydrophobic core with I239, Y256, L260 and L270 of the GatA subunit (Figure 4B). These hydrophobic residues from GatF are highly conserved among the fungal AdTs (Supplementary Figure S3). On the other hand, the following loop and α2 helix form electrostatic interactions with GatA (Figure 4C). The main chain carbonyl group of R48 of GatF forms a salt bridge with H245 of GatA (Figure 4C). In the α2 helix, H54 of GatF forms a salt bridge with E246 of GatA, while Y56 of GatF hydrogen bonds with N280 of GatA (Figure 4C). However, as compared to the hydrophobic core in the β1 strand and the α1 helix, these hydrophilic residues are not well conserved among the fungal AdTs (Supplementary Figure S3). Intriguingly, the side chain of H54 is disordered in the apo form structure, while all of the other hydrophilic and hydrophobic interactions between GatF and GatA are preserved in both the Gln-bound and free forms. Notably, H54 is located in the vicinity of the glutaminase pocket in the Gln-bound form, implying that its stability depends on Gln-binding. Given that the interface is adjacent to the glutaminase site (Figure 4A), the interaction between the GatF NTD and GatA seems to play a key role in stabilizing the glutaminase site for effective glutamine hydrolysis.

### Functional analyses for N-terminal deletion mutants

**Figure 5.**
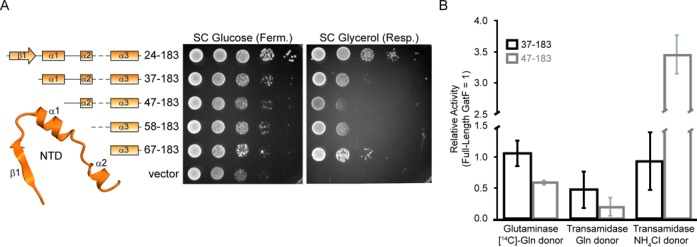
Functional analyses of the GatF NTD. (**A**) Secondary structures of GatF mutants are shown (left). Growth phenotypes of GatF mutants on fermentative (SC Glucose) or respiratory (SC Glycerol) media (right). (**B**) Quantification of the activities of the GatFAB_F37–183_ and GatFAB_F47–183_ mutants relative to the full-length GatFAB. Glutaminase activity as well as transamidase activities with glutamine or NH_4_Cl as the amide donor are shown for GatF mutants relative to the activity of the full-length GatFAB. Results are the average of three experiments, with error bars representing standard deviation (See Supplementary Figure S5).

To verify *in vivo* the functional properties of the NTD, we tested a series of N-terminal mutants (GatF_24–183_, GatF_37–183_, GatF_47–183_, GatF_58–183_ and GatF_67–183_) for their ability to complement the deletion of the WT *gatF* gene (Figure 5A). GatF_24–183_ lacks the N-terminal MTS but retains the entire NTD, whereas GatF_67–183_ completely lacks the NTD and consists of only the GatC-like portion of GatF. The other variants, GatF_37–183_, GatF_47–183_ and GatF_58–183_, partially lack the NTD. Since all of the GatF mutants lack the endogenous N-terminal putative MTS, we fused the MTS of MDH to the N-terminus of each GatF mutant as *in vivo* analyses of GatB mutants.

The GatF_24–183_ mutant did not exhibit any growth defects in either fermentation or respiration, revealing that the MTS of MDH allows mitochondrial import of GatF (Supplementary Figure S1B). As compared to the pseudo-wild-type GatF_24–183_ variant, the progressive NTD deletions in the GatF_37–183_, GatF_47–183_ and GatF_58–183_ mutants impacted growth on respiratory medium, but did not completely prevent the use of the respiratory metabolism (Figure 5A). Strangely, the mutant that lacked the entire NTD (GatF_67–183_) grew slightly better than that bearing the loop connecting the α2 and α3 helices (GatF_58–183_). These results indicated that the NTD is highly important for the enzymatic activity of GatFAB, but the GatC-like domain can still assemble a functional GatFAB AdT that forms sufficient amounts of Gln-_m_tRNA^Gln^ *in vivo* (Figure 5A). Unexpectedly, the Δ*gatF* strains and the GatF mutants displayed mild growth defects in fermentation, suggesting the participation of GatF in other pathways during fermentative growth.

To explore the functional role of the NTD in tRNA-dependent Gln synthesis, we generated *E. coli* strains expressing the GatFAB mutants with sequential N-terminal deletions of GatF. Immunoblot analysis revealed that all of the truncated mutants showed marked defects in their heterologous expression levels as compared to the pseudo-wild-type GatFAB_F24–183_ variant (Supplementary Figure S4A). When purified, all three subunits were equimolar and robustly detected in GatFAB_F37–183_ and GatFAB_F47–183_ variants on coomassie blue-stained protein gel (Supplementary Figure S4B). The minor bands between GatF and GatA were contaminants from the host cell. Thus, both GatF_37–183_ and GatF_47–183_ are still capable of assembling a GatFAB heterotrimer. However, in the fractions of GatFAB_F58–183_ and GatFAB_F67–183_ variants, all three subunits were difficult to detect (Supplementary Figure S4B). These results showed that the expression was drastically decreased with the shortening of the NTD, implying that the NTD contributes to stability of the GatA subunit to prevent its degradation in the cell extract.

Due to the especially low expression levels, we were unable to purify the GatFAB_F58–183_ and GatFAB_F67–183_ mutants. However, we obtained sufficient quantities of the GatFAB_F37–183_ and GatFAB_F47–183_ mutants, which retain the α2 helix but partially or completely lack the hydrophobic core, for enzymatic activity measurements.

In agreement with its participation in GatA folding, the GatFAB_F37–183_ and GatFAB_F47–183_ mutants showed reduced activities in both GatA-mediated glutamine-hydrolysis and GatB-mediated transamidation when compared to that of the full-length (Figure 5B and Supplementary Figure S5). When the glutaminase reaction was bypassed by using ammonium chloride as the ammonia donor, a shorter NTD seemed to favor transamidation (Figure 5B and Supplementary Figure S5). Notably, the difference in transamidation activity with free ammonia as the donor between the GatFAB_F37–18_ mutant and the GatFAB_F47–183_, which has lost the hydrophobic cluster, highlights the importance of this cluster in modulating the folding of the glutaminase site. In agreement with our observations of the GatFAB structure, the biochemical data therefore further suggest that the NTD is strongly linked to the glutaminase activity, likely by playing a key role in stabilizing the GatA core structure, but possibly also by affecting the use of free ammonia. Meanwhile, the effect on transamidation could be caused by unstable folding of the deletion mutants affecting the trimeric assembly, and/or be a consequence of the reduction in glutaminase activity.

## DISCUSSION

In many eukaryotes, Gln-_m_tRNA^Gln^ is generated by the transamidation pathway employing a ND-GluRS and an AdT. Although the orthologs encoding catalytic subunits such as GatA and GatB can be easily identified in the majority of the eukaryotic genomes, the third auxiliary subunit shows more sequence variation (10).

While the GatC subunit is used in most eukaryotes analyzed so far, fungi utilize the GatF subunit. The reason why fungi evolved this subunit, with its NTD extension domain, remains unknown. Our structural analyses revealed that GatF adopts an extended structure similar to that of GatC by surrounding the main catalytic body. The portions of the GatAB interface that are conventionally stabilized by GatC are also stabilized by the GatC-like portion of GatF, albeit by different mechanisms on the local scale (Supplementary Figure S2). On the larger scale, the main difference is the additional interface between GatA and the NTD of GatF. GatF therefore assures the structural role of GatC in addition to its own, despite the lack of sequence homology. Such a role in GatA stabilization is observed in the bacterial GatC. Previous work proposed that the bacterial GatC subunit is required for folding of the GatA subunit (31). Our current study can provide insights into the role of the small, non-catalytic subunit of the trimeric AdTs. GatA is unstable, and therefore needs to be coexpressed with non-catalytic subunits, GatC or GatF. In bacteria, the ring structure of GatC is sufficient to retain the GatA structure. However, in yeast mitochondria, the GatA subunit may be more unstable and need extensive interactions provided by the NTD of GatF. Our functional analyses revealed that the NTD contributes to the GatA glutaminase activity, suggesting that the yeast organellar GatF subunit is both a glutaminase scaffolding peptide and an assembly factor. The increased rate of transamidation for GatFAB_F47–183_ when NH_4_Cl was the ammonia donor hints at a role in controlling the use of free ammonia.

Given that the NTD-deprived GatF strains can still respire, despite their impaired ability to efficiently generate NH_3_ by Gln deamination, we considered that a Δ*gatF* mutant should be rescued by the overexpression of a bona fide mitochondrial *gatC* gene. A previous study reported that a respiration-defective mutant of the GatB gene was complemented by the expression of *Bacillus subtilis* GatB fused with an MTS, suggesting that a chimeric GatCAB has sufficient activity *in vivo* (32). However, the overexpression of the human GatC fused to the MDH MTS did not rescue the growth of the Δ*gatF* KO strain (Supplementary Figure S6). This result suggested that yeast GatA and GatB are not able to form an active chimeric AdT even with another organellar GatC, perhaps due to the structural differences in the internal loops (Supplementary Figure S2). Another possibility would be that GatF may play additional roles in mitochondria that are not supported by GatC, such as signal transduction or adaptation to the mitochondrial environment, in addition to its primary function as a GatA–GatB assembly factor.

**Figure 6.**
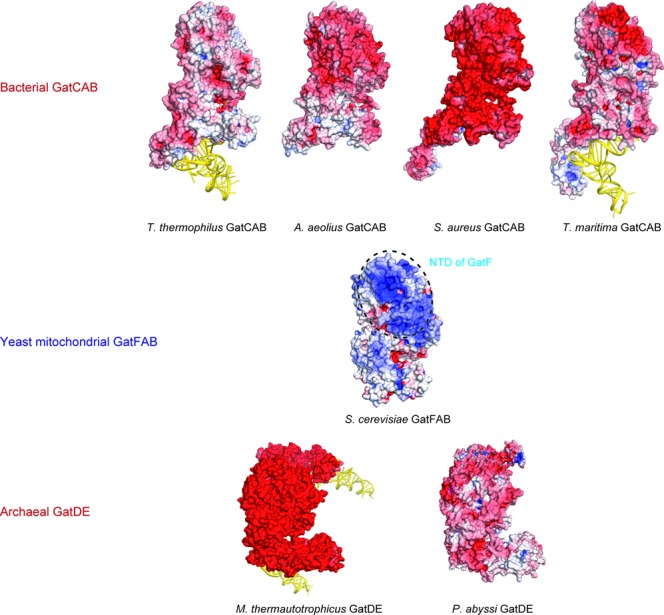
Electrostatic surface potential models of tRNA-dependent amidotransferases. Bacterial GatCABs (PDB IDs: 3KFU for *T. thermophilus* GatCAB-tRNA^Asn^ complex, 3H0R for *A. aeolius* GatCAB, 3IP4 for *S. aureus* GatCAB and 3AL0 for *T. maritima* GatCAB-tRNA^Gln^ complex) are shown on the upper lane. *S. cerevisiae* GatFAB (this study) is shown on the center of the figure. The NTD of GatF is indicated by the dashed circle. Archaeal GatDEs (PDB IDs: 2D6F for *M. thermautotrophicus* GatDE-tRNA^Gln^ complex and 1ZQ1 for *P. abyssi* GatDE) are shown on the lower lane. Electrostatic surface potential is calculated by the program APBS (36).

Intriguingly, the electrostatic potentials of the solvent-accessible surface models are quite different between the bacterial and yeast mitochondrial AdTs (Figure 6). While all reported bacterial GatCAB structures, *S. aureus, A. aeolicus, T. thermophilus and T. maritima* GatCABs, are negatively charged (14–18), the yeast GatFAB has positively charged patches spread over the GatF and GatA subunits. Notably, all reported archaeal GatDE AdT structures, *Pyrococcus abyssi* and *Methanothermobacter thermautotrophicus* GatDEs, are also negatively charged (33,34). These differences may be an adaptation to the physiological environment, or relevant to the localization in organelles. Previous reports indicated that the GatF subunit can be isolated from the inner membrane, and that it likely faces the matrix (30), although GatF does not have any specific domain for anchoring to the phospholipid membrane (35). The presence of the positively charged patch provides a possible explanation for the anchoring of GatFAB to the inner membrane through electrostatic interactions with negatively charged phospholipids. However, it remains unclear whether GatF is directly anchored to the inner membrane or if it requires other adapter molecules for anchoring. The considerable difference in electrostatic surface potential between fungal AdT and those previously described could also be exploitable with respect to the development of inhibitory compounds specific to fungi. It may also be related to the impaired use of free ammonia as an amide donor in the full-length enzyme compared to an NTD truncated mutant.

**Figure 7.**
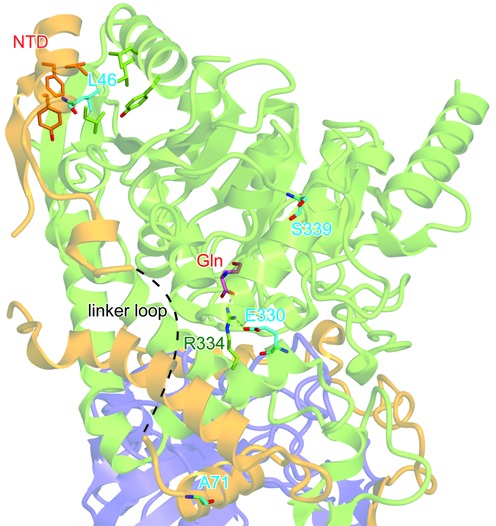
Structural mapping of respiration-defective mutations. GatFAB is shown as in Figure 1. The residues associated with the respiration-defective phenotypes are shown as cyan sticks. The bound Gln molecule is shown as pink sticks.

A recent study reported that the L46S and A71G double mutations in the yeast GatF subunit cause a temperature-sensitive (ts) respiratory phenotype (30). This ts mutant strain accumulated defective forms of the *atp8* and *cox2* gene products due to significantly decreased Gln-_m_tRNA^Gln^ formation (30). The present structure revealed that these residues are located on the NTD (Figure 7). L46 participates in the conserved GatF hydrophobic core contacts between GatF and GatA. A71 is located in between the NTD and the GatC-like portion of GatF. Based on the present structure, the L46S and A71G double mutations may weaken the hydrophobic interaction in the GatF–GatA interface, by increasing the flexibility of the disordered linker loop and/or destabilizing the GatA structure, thereby impairing the glutaminase activity. Thus, the structural mapping of these mutations agrees well with our present work, showing that the enhanced stabilizing interactions of the NTD are critical for the enzymatic activity of GatA, and thus the efficiency of mitochondrial translation.

Moreover, the GatA E330K and S399F double mutations also lead to respiration-defective phenotypes, which could be suppressed by the overexpression of GatF (30). Notably, these mutations are located in the vicinity of the glutaminase active site (Figure 7). E330 is highly conserved and interacts with R334, which recognizes the γ-carbonyl group of the Gln substrate molecule, indicating that the E330K mutation can disrupt this interaction and reduce the affinity for Gln. On the other hand, S399 is not well conserved, but the S399F mutation may reduce the glutaminase activity by increased steric hindrance due to the aromatic side chain. Therefore, our structural mapping revealed that the mutations of both the glutaminase site of GatA and the NTD of GatF display similar functional phenotypes, confirming the existence of a tight functional and structural relationship between the glutaminase activity and the presence of the NTD.

To conclude, our work describes the structure of the yeast mitochondrial GatFAB AdT displaying a new type of subunit, GatF. We proved that the yeast GatFAB AdT employs the conserved glutaminase and AdT active sites connected by a molecular tunnel. GatF is composed of the GatC-like portion and the NTD. While the GatC-like portion encircles the GatA–GatB contact area, the NTD forms extended interactions with the GatA core domain. Moreover, the structure-based functional analyses suggest the NTD is a trans-acting scaffolding peptide for GatA. Our findings shed a new light on the architecture and function of the mitochondrial tRNA-dependent AdT, as well as on glutamine decoding in mitochondria.

## ACCESSION NUMBERS

The atomic coordinates are deposited in the Protein Data Bank (PDB ID codes: 4N0H and 4N0I).

## SUPPLEMENTARY DATA

Supplementary Data are available at NAR Online.

SUPPLEMENTARY DATA
