# Supplementary material for: Crystal structure of Saccharomyces cerevisiae mitochondrial GatFAB reveals a novel subunit assembly in tRNA-dependent amidotransferases
Source: Nucleic Acids Res. 2014 Apr 1;42(9):6052–63. doi: 10.1093/nar/gku234 (PMC4027206; doi:10.1093/nar/gku234)
Supplement: SUPPLEMENTARY DATA [file supp_42_9_6052__index.html]

Crystal structure of Saccharomyces cerevisiae mitochondrial GatFAB reveals a novel subunit assembly in tRNA-dependent amidotransferases — Crystal structure of Saccharomyces cerevisiae mitochondrial GatFAB reveals a novel subunit assembly in tRNA-dependent amidotransferases — Crystal structure of Saccharomyces cerevisiae mitochondrial GatFAB reveals a novel subunit assembly in tRNA-dependent amidotransferases — SUPPLEMENTARY DATA 

# Crystal structure of *Saccharomyces cerevisiae* mitochondrial GatFAB reveals a novel subunit assembly in tRNA-dependent amidotransferases

## SUPPLEMENTARY DATA

**Files in this Data Supplement:**

- SUPPLEMENTARY DATA
