# Supplementary material for: Crystal structure of Saccharomyces cerevisiae mitochondrial GatFAB reveals a novel subunit assembly in tRNA-dependent amidotransferases
Source: Nucleic Acids Res. 2014 Apr 1;42(9):6052–63. doi: 10.1093/nar/gku234 (PMC4027206; doi:10.1093/nar/gku234)
Supplement: SUPPLEMENTARY DATA [file supp_gku234_nar-03680-f-2013-File009.pdf]

Supplementary data

Crystal structure of *Saccharomyces cerevisiae* mitochondrial GatFAB reveals a novel subunit assembly in tRNA-dependent amidotransferases

Yuhei Araiso[1], Jonathan L. Huot[1], Takuya Sekiguchi[2], Mathieu Frechin[3], Frédéric Fischer[4], Ludovic Enkler[1], Bruno Senger[1], Ryuichiro Ishitani[2], Hubert D. Becker[1,]* and Osamu Nureki[2,]*

[1] Unité Mixte de Recherche 7156 Génétique Moléculaire Génomique Microbiologie, Centre National de la Recherche Scientifique, Université de Strasbourg, F-67084 Strasbourg, France

[2] Department of Biophysics and Biochemistry, Graduate School of Science, The University of Tokyo, Bunkyo-ku, 113-0033 Tokyo, Japan

[3] Institute of Molecular Life Sciences, University of Zurich, CH-8057 Zurich, Switzerland

[4] Unité Propre de Recherche Architecture et Réactivité de l'ARN, Centre National de la Recherche Scientifique, Institut de Biologie Moléculaire et Cellulaire, Université de Strasbourg, F-67084 Strasbourg, France

* To whom correspondence should be addressed. Tel: +81 (0)3 5841 4392; Fax: +81 (0)3 5841 8057; Email: nureki@biochem.s.u-tokyo.ac.jp

Correspondence may also be addressed to Hubert D. Becker. Tel: +33 (0)3 68 85 14 70; Fax: +33 (0)3 68 85 13 65; Email: h.becker@unistra.fr

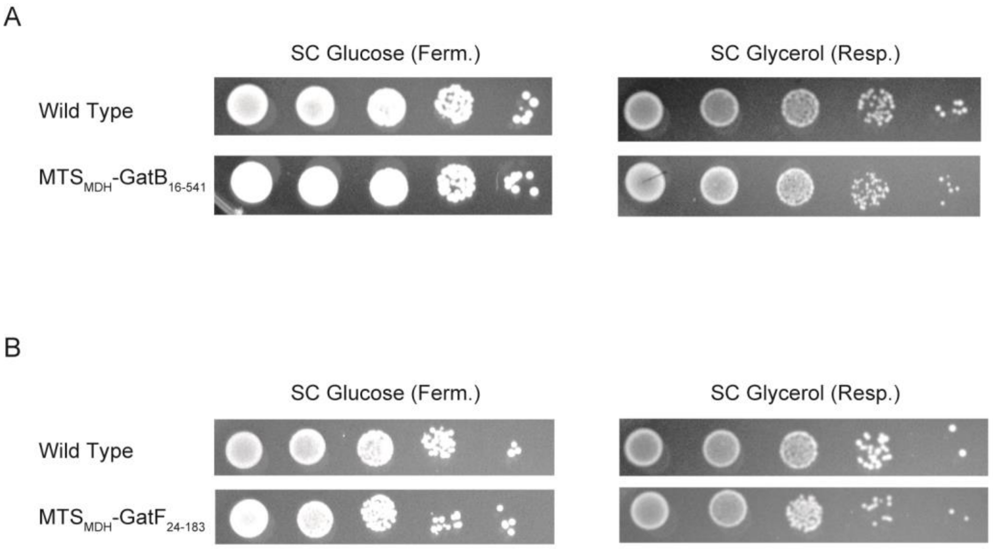

Figure S1. Yeast complementation of the RS453 wild type strain and the GatB$_{16-541}$ (A) and GatF$_{24-183}$ (B) mutants fused to MTS of MDH.

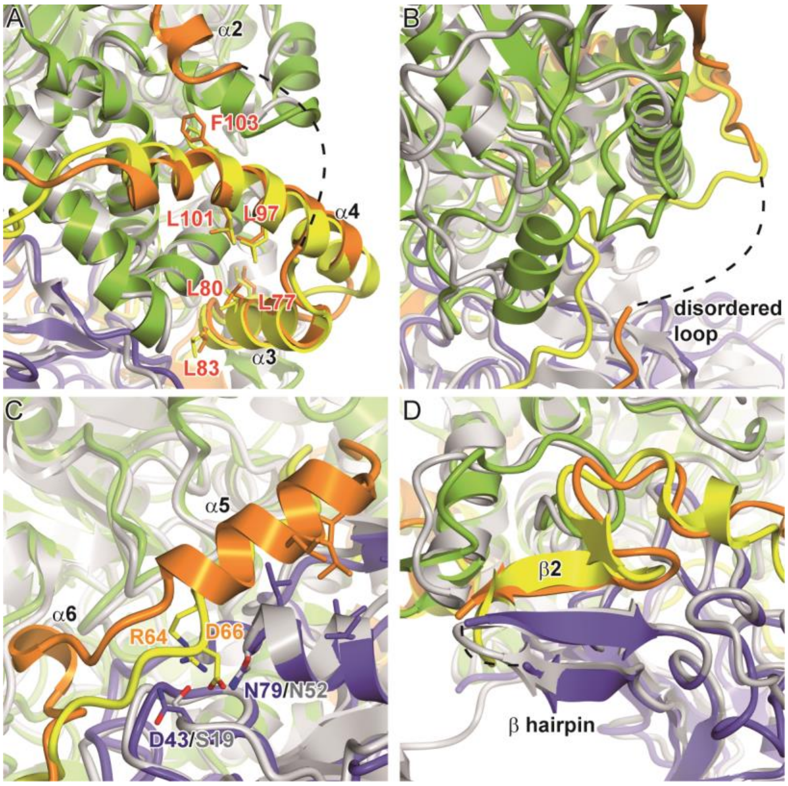

Figure S2. Interactions between GatAB and the GatC-like portion of GatF.

The crystal structure of *S. aureus* GatCAB is superposed onto that of GatFAB. GatFAB is colored as in Figure 1. *S. aureus* GatAB and GatC are colored gray and purple, respectively. (A) The conserved helical bundle. The conserved hydrophobic residues between GatF and GatC are shown as yellow and purple sticks, respectively. (B) The disordered loop followed by the helical bundle. (C) The insertion helices α5 and α6. The residues involving hydrophobic interactions for anchoring α5 helix are shown as yellow and green sticks. The conserved residues forming a hydrogen bond network in bacterial GatC are shown as purple, green and gray sticks. (D)The C-terminal β2 strand forming an antiparallel β-sheet with a β hairpin of GatB.

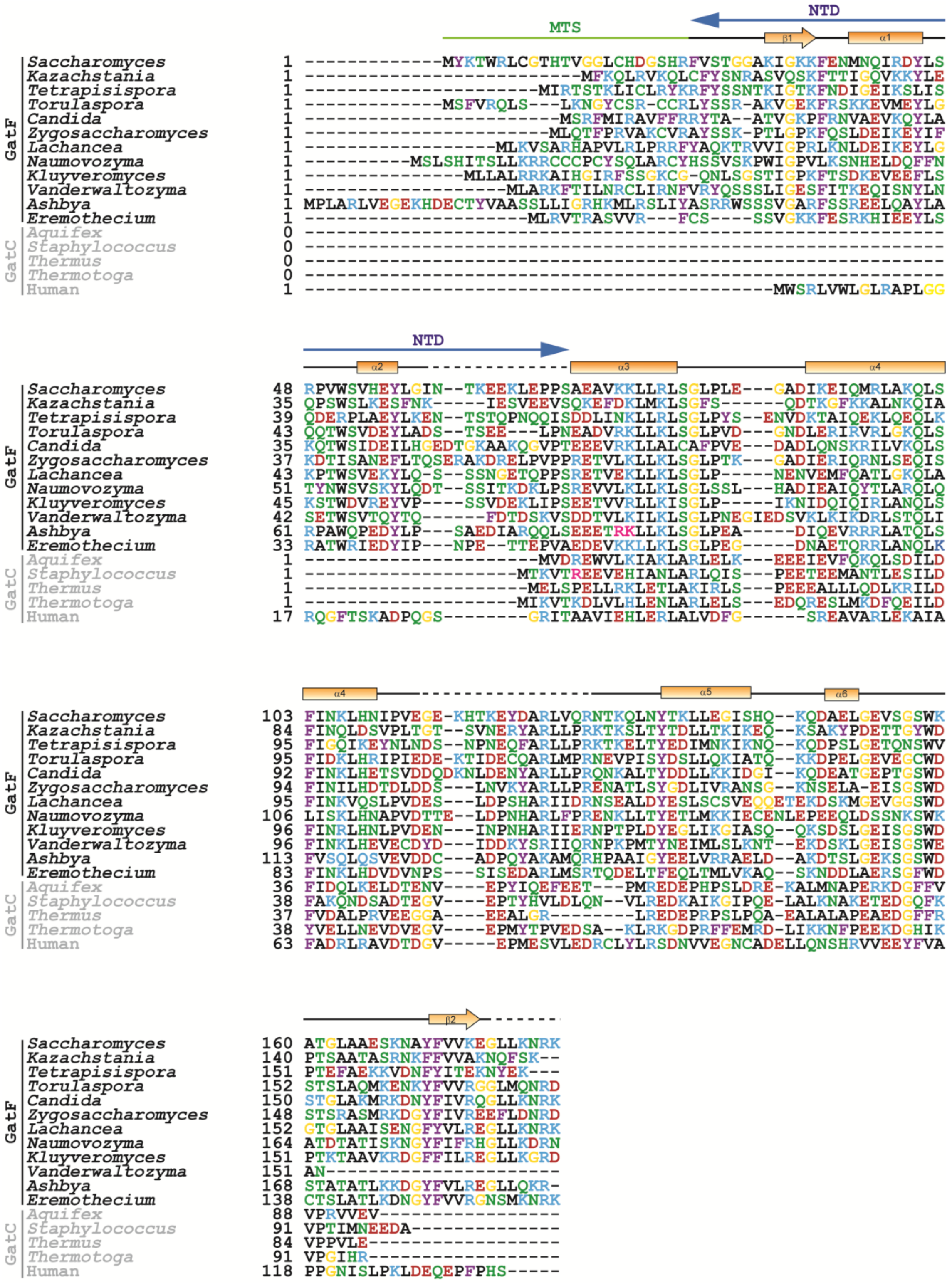

Figure S3. Sequence alignment of *S. cerevisiae* GatF with GatF and GatC.

The MTS, NTD and secondary structure of *S. cerevisiae* GatF are illustrated.

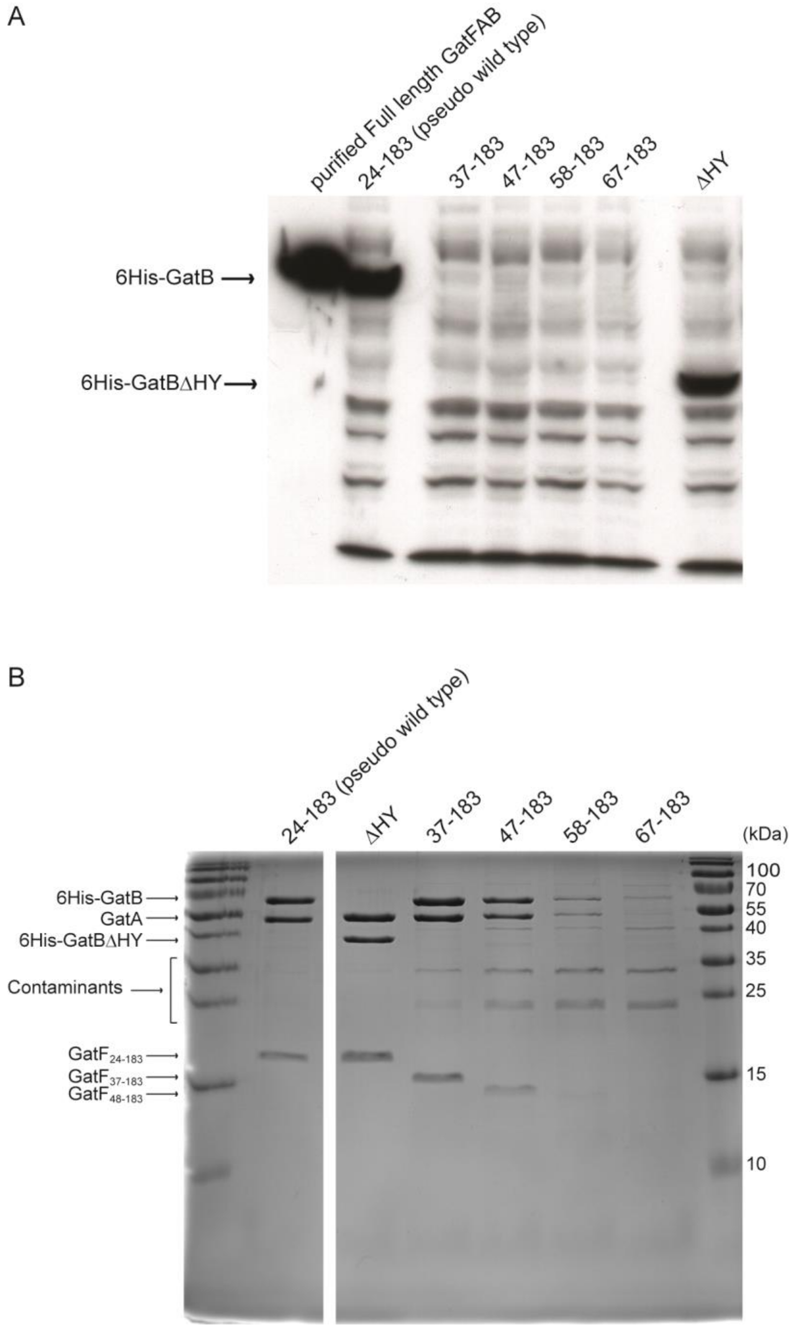

Figure S4.Expression and purification of the GatFAB mutants.

(A) Western blot quantification of the expression levels of GatFAB mutants in *E. coli* total protein extract by anti-His tag antibody. 6His tag is fused to the C-terminus of GatB in each construction. (B) Coomassie blue-stained protein gel of the GatFAB mutants, purified by MagneHis (Promega).

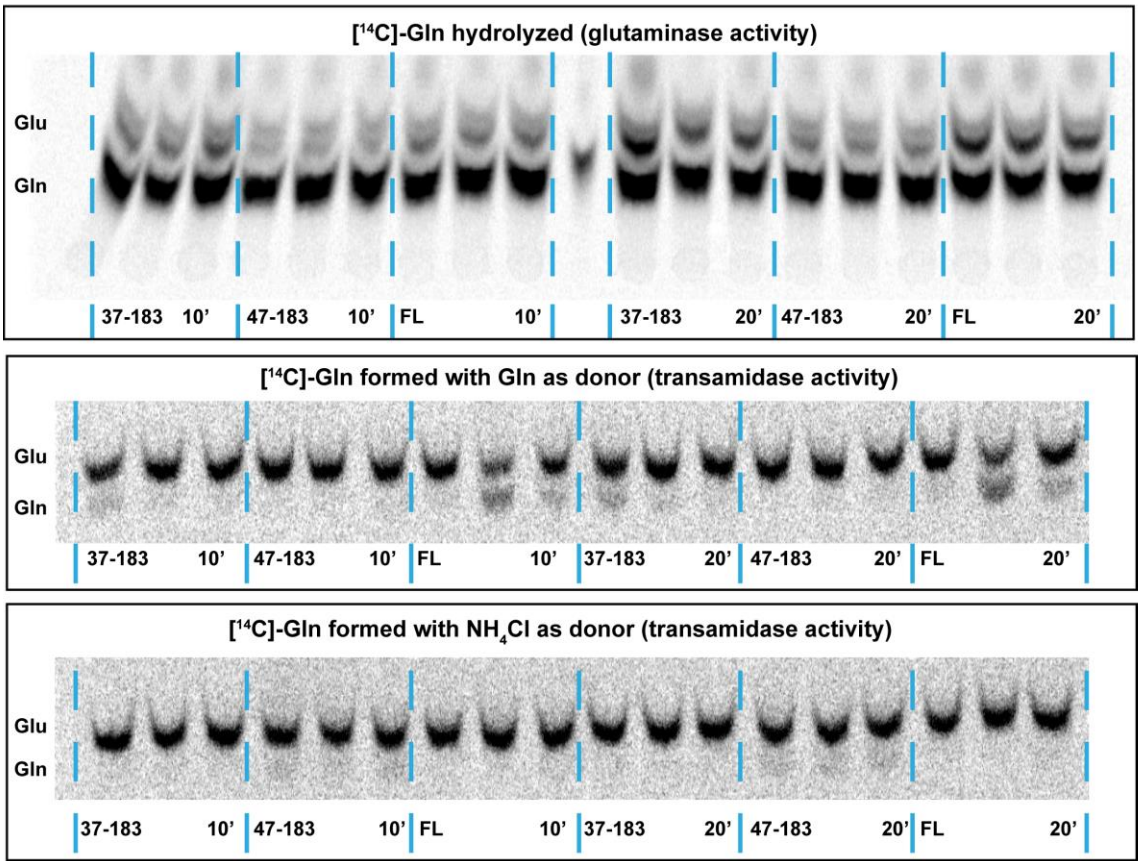

Figure S5. Thin-layer chromatography activity assays for GatF NTD deletion mutants.

Three reactions were performed for each of the three activities tested: Glutamine hydrolysis, transamidation with Gln donor and transamidation with NH_4Cl donor. Time points were taken at 10 and 20 minutes. Relative activities were obtained by dividing the value of each of three points for a mutant at a given time for a given reaction (*e.g.*, glutaminase, 37-183, 10’) by the average value of the three corresponding full length enzyme measurements (*e.g.*, glutaminase, FL, 10’). Relative activities at 10’ and 20’ were combined and the averages of those six relative activities for each of the two mutants in the three conditions tested are presented in Figure 5B. The standard deviation of each group of six time points was used for the error bars.

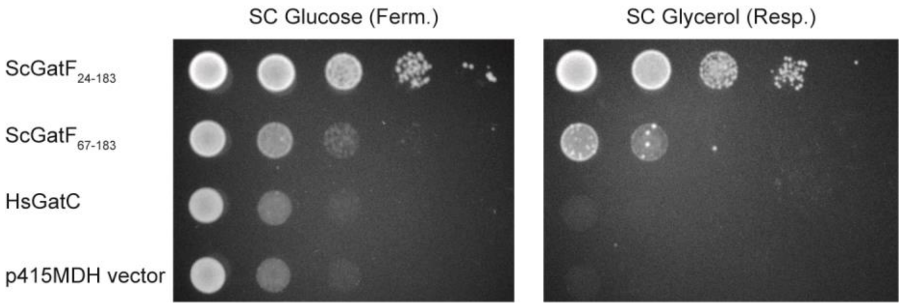

Figure S6. Yeast complementation of the NTD-deprived GatF mutant (GatF$_{67\text{-}183}$) and human GatC.
